# Supplementary material for: Liquid Biopsies in Lung Cancer
Source: Cancers (Basel). 2023 Feb 23;15(5):1430. doi: 10.3390/cancers15051430 (PMC10000706; doi:10.3390/cancers15051430)
Supplement: Supplementary file 1 [file cancers-15-01430-s001.zip › cancers-2212626-supplementary.pdf]

Supplemenatry Materials: Liquid Biopsies in Lung Cancer

Marcel Kemper, Carolin Krekeler, Kerstin Menck, Georg Lenz, Georg Evers, Arik Bernard Schulze and Annalen Bleckmann

Table S1. Overview of studies, partly using liquid biopsy in mutational diagnostics, cited in this review.

Study	Details	NCT Number	Status	*n*	Stages (UICC)	Histological Types	(Diagnostic) Result
LIQUIK	Observational	NCT04703153	Active, recruiting	200 (estimated enrollment)	IV	Non-squamous NSCLC	Ongoing, no update
AURA 3	Phase III	NCT02151981	Active, not recruiting	419	III, IV	NSCLC with EGFR mutation with prior EGFR-directed therapy	Superiority of osimertinib treatment compared to platinum-containing cytotoxic treatment after progression on first-line TKI therapy regarding PFS, ORR and adverse events
FLAURA2	Phase III	NCT04035486	Active, not recruiting	556	III B, IIIC, IV	NSCLC with EGFR mutation, no prior therapy	Superiority of osimertinib compared to comparator-TKI (gefitinib, erlotinib) regarding OS and exposure time
APPLE	Phase II	NCT02856893	Active, not recruiting	156	IV	NSCLC (adenocarcinoma only) with plasma-confirmed EGFR mutations Del19 or L858R	Ongoing, no update
MELROSE	Phase II	NCT03865511	Active, recruiting	66 (estimated enrollment)	IIIB/IV	NSCLC (adenocarcinoma only), treatment-naïve with at least one of TKI-sensitivity-harboring EGFR mutations (Ex19 deletions or L858R)	Ongoing, no update
AURA	Phase II	NCT01802632	terminated	603	IIIB/IV (advanced, non-operable)	EGFR mutated NSCLC with progression on single-agent TKI	Osimertinib shows efficacy (PFS 9.6 months) and high ORR (61%) in EGFR T790M mutated NSCLC
B-FAST	Phase II/III	NCT03178552	Active, recruiting	1000 (estimated enrollment)	III (advanced, not amenable to treatment with combined chemoradiation)/IV	NSCLC<br>Cohort A: ALK pos<br>Cohort B: RET pos<br>Cohort C: bTMB pos.<br>Cohort D: ROS1+<br>Cohort E: BRAF V600<br>Cohort F: Exon 20 +<br>Cohort G: KRASG12C	For Cohort A: Clinical application of blood-based NGS as a method to inform clinical decision making in ALK-positive NSCLC
VISION	Phase II	NCT02864992	Active, not recruiting	337	IIIB/IV	NSCLC (all types, including sarcomatoid and squamous) with METex14 skipping mutation	Tepotinib treatment in METex14 skipping: ORR 48% (liquid-based assessment) vs. 50% (tissue-based), mDOR 11.1 months independent from prior treatment
Lu et al.	Phase II	NCT02897479	Active, not recruiting	76	IIIB/IV	Pulmonary sarcomatoid carcinoma and other NSLCL harboring	Savolitinib in METex14 skipping alterations leads to ORR to 49.2%, baseline positivity in

						METex14 skipping mutation	ctDNA sequencing had worse PFS and OS but higher ORR
LIBRETTO	Phase I/II	NCT03157128	Active, recruiting	875 (all arms, estimated enrollment)	Locally advanced or metastastic solid tumors	Medullary thyroid cancer, NSCLC, other solid tumors with RET mutation/fusion/alteration	Selpercatinib treatment in RET pos. NSCLC leads to an ORR of 64% (85% in untreated patients), mDOR 17.5 months; high activity in brain metastases (intracranial response rate 91%)
ARROW	Phase I/II	NCT03037385	Active, not recruiting	589 (all arms)	Locally advanced or metastastic solid tumors	Medullary thyroid cancer, NSCLC, other solid tumors with RET mutation/fusion/alteration	Praseltinib treatment in RET pos. NSCLC leads to an ORR of 53% (70% in untreated patients), 3 complete responses
CodeBreak	Phase I/II	NCT03600883	Active, not recruiting	713 (all arms)	Locally advanced or metastastic solid tumors	All entities with pathologically confirmed KRAS p.G12C mutation	Sotorastib treatment in NSCLC with KRAS p.G12C mutation leads to an ORR in 37.1% besides multi-lineage pre-treatment and disease control rate (DCR) in 80.6% of all patients, mPFS 6.8 months, mOS 12.5 months
Code-Break200	Phase III	NCT04303780	Active, not recruiting	345	Locally advanced and unresectable or metastatic NSCLC	Locally advanced and unresectable or metastatic NSCLC, pathologically confirmed KRAS p.G12C mutation	Sotorasib significantly improves PFS and has a more favorable safety profile vs. docetaxel
ClinCode-Break200	Phase Ib/II	NCT04185883	Active, recruiting	1,143 (estimated enrollment)	Locally advanced or metastatic malignancies	All entities with pathologically confirmed KRAS p.G12C mutation	Higher rates of grade III and IV adverse events combining sotorasib and immunotherapeutics, dose escalation ongoing
MYSTIC	Phase III	NCT02453282	Active, not recruiting	1148	IV	NSCLC, no sarcomatoid variant	no superiority of tremelimumab and durvalumab vs. cytotoxic treatment in first-line setting, optimal benefit at bTMB >20 mut/mb
POPLAR trial	Phase II	NCT01903993	completed	287	IIIB, IV	NSCLC	Significant improval of OS with atezolizumab treatment compared to docetaxel
OAK trial	Phase III	NCT02008227	completed	1,225	IIIB, IV	NSCLC	Improvement of survival of atezolizumab vs. docetaxel after platinum-based chemotherapy
B-F1RST	Phase II	NCT02848651	completed	153	IIIB, IV	NSCLC	bTMB ≥ 16 was associated with higher ORR and longer OS compared to bTMB <16 under atezolizumab treatment

Table S2. Use of liquid biopsies in lung cancer screening, post-treatment surveillance and therapy response prediction.

Study	Details	NCT number	Status	*n*	Subjects	Further Details	(Diagnostic) Result
DETECT-A	Prospective interventional	-	Completed	10,006	Healthy women	-	Screening via blood test revealed 26 cancer cases, confirmed by PET-CT

SUMMIT	Observa-tional	NCT03934866	Active, not re-cruiting	13,035	Smoker/high lung cancer risk	30 pack years (py) or lung cancer risk ≥1.3% as-sessed by PLCOm2012	Timely reporting of 645 (5.6%) LD-CT findings during 5 days is feasible
AIR	Prospective, observa-tional	NCT02500693	Active, not re-cruiting	614	Smokers and ex-smokers (≥ 30 py)	With COPD	3% of patients were detected with prevalent lung cancer at first screening, and 4% with in-cident lung cancer during fol-low-up
SUPE_R	RCT	NCT03740126	Active, not re-cruiting	750	NSCLC pa-tients	I-III, eligible for definitive treatment	Surveillance with liquid biopsy and PET-CT after definitive treatment: Ongoing, no update
ORACLE	Prospective, observa-tional	NCT05059444	Active, re-cruiting	1,000	Patients with solid tumors	treatment in curative in-tent	Feasibility of ctDNA testing in observing residual tumor cells: Ongoing, no update
TRACERX	Prospective, observa-tional	NCT01888601	Active, not re-cruiting	814	NSCLC	Stage I-IIIA	Multi-regional exome and RNA-seq mapped with deep learning elucidates constraints that may shape the emerge of immune-evading subclones and aggressive phenotypes
LIBER-TYLUNG	Prospective, observa-tional	NCT04790682	Active, re-cruiting	300	NSCLC	Stage IV	Feasibility of liquid biopsy to predict responses to first-line immunotherapy
